# The Size of the Human Proteome: The Width and Depth

**DOI:** 10.1155/2016/7436849

**Published:** 2016-05-19

**Authors:** Elena A. Ponomarenko, Ekaterina V. Poverennaya, Ekaterina V. Ilgisonis, Mikhail A. Pyatnitskiy, Arthur T. Kopylov, Victor G. Zgoda, Andrey V. Lisitsa, Alexander I. Archakov

**Affiliations:** Institute of Biomedical Chemistry, Moscow 119121, Russia

## Abstract

This work discusses bioinformatics and experimental approaches to explore the human proteome, a constellation of proteins expressed in different tissues and organs. As the human proteome is not a static entity, it seems necessary to estimate the number of different protein species (proteoforms) and measure the number of copies of the same protein in a specific tissue. Here, meta-analysis of neXtProt knowledge base is proposed for theoretical prediction of the number of different proteoforms that arise from alternative splicing (AS), single amino acid polymorphisms (SAPs), and posttranslational modifications (PTMs). Three possible cases are considered: (1) PTMs and SAPs appear exclusively in the canonical sequences of proteins, but not in splice variants; (2) PTMs and SAPs can occur in both proteins encoded by canonical sequences and in splice variants; (3) all modification types (AS, SAP, and PTM) occur as independent events. Experimental validation of proteoforms is limited by the analytical sensitivity of proteomic technology. A bell-shaped distribution histogram was generated for proteins encoded by a single chromosome, with the estimation of copy numbers in plasma, liver, and HepG2 cell line. The proposed metabioinformatics approaches can be used for estimation of the number of different proteoforms for any group of protein-coding genes.

## 1. From Human Genome to Human Proteome

Genome sequencing [1] deciphered the number of protein-coding genes, establishing an initial estimation of complexity associated with human molecular biology. The next step is to obtain similar benchmarks at the proteome level. Two recent articles described creation of a draft of the human proteome [2, 3]. Nevertheless, considerable efforts are still required for exploring the space (or size) of the human proteome, as a compulsory constellation of molecular profiles of different tissues and organs. The human proteome is quite a dynamic entity [4] and this property should be considered in two dimensions. The first is to estimate the number of different protein types (proteome width), as well as measure protein copies number in particular tissues (proteome depth).

Following the hypothesis of “one gene = one protein,” there should be at least ~20,000 nonmodified (canonical) human proteins. Taking into account products of alternative splicing (AS), those containing single amino acid polymorphisms (SAPs) arising from nonsynonymous single-nucleotide polymorphisms (nsSNPs), and those that undergo PTMs [4, 5], as many as 100 different proteins can potentially be produced from a single gene. Of the many different terms proposed to describe protein variants [6], here, we chose “protein species” [7] or “proteoforms” [6].

Experimental validation of protein species is limited by the analytical sensitivity of proteomic technology. This means that the sensitivity of the technology determines the ability to detect rare protein species. This limitation originates from the basic difference between genomics and proteomics [8]. Genomics relies upon PCR [9] to amplify DNA or RNA molecules in a biological sample to concentrations above the detection threshold. However, there currently exists no comparable high-throughput technology capable of multiplying the copies of a single protein [8].

The 100% coverage of protein sequence using bottom-up MS is not attainable; thus, it is impossible to detect all potential protein species expressed from the same gene. Generally, proteome investigations are focused on the* master proteins* resembling at least one of the many possible proteoforms, coded by the gene and containing at least one MS-detectable proteotypic peptide. The sequence could be modified or nonmodified, so this means that the master protein could be present as a single protein or as a set of proteins.* Master proteome* of a single chromosome is the result of the identification and measurement of all master proteins encoded by the chromosome and expressed in the selected type of biological material. For experimental validation of proteoforms, the targeted MS analysis should be performed in order to probe candidate sequence alteration. The bioinformatic analysis of the diversity of protein species was anticipated to create the backbone for the future experimental exploration of the proteome space.

## 2. How Many Different Proteins Are Necessary to Support Human Function?

The number of different proteins comprising the human proteome is a core proteomics issue. Researchers propose numbers between 10,000 [10] and several billion [6] different protein species. Here, we describe the theoretical prediction for the number of different proteoforms that might arise from AS, SAP, or PTM events.

The data was derived from neXtProt, which contains only human proteins and their modifications and sequence features [11]. The neXtProt annotation of AS, SAP, and PTM originated from biocuration of the data from repositories, literature, and prediction tools. Information on possible protein sequence variability is represented as the number of AS variants, nsSNPs/SAPs, and PTMs per gene.

Our assumption was that database extension and annotation are a constituent process, whose rate is mostly limited by the number of the researchers and annotators around the world. The rate is slightly dependent on the capacity of communication channel and information accessibility, as these were not changed too much for 10–15 years for the needs of PubMed or UniProt users. Therefore, the extension of the number of annotations in a certain database would generally be affected by the technology achievements, gained by increasing the sensitivity/throughput of the bioanalytical method.

From the above, we proposed that the volume of representative data uploaded to UniProt [12] each year from 2005 was sufficient to calculate the average number of protein variants per one gene and the numbers for each type of variation. Interestingly, since 2010, the average number of modifications per one gene has remained nearly the same, despite the continuous increase in reviewed annotations. The average number of modifications specifically by AS (40% reviewed annotations out of all data records), SAP (60% reviewed annotations), or PTM (37%) remains almost unchanged.

The saturation in the number of annotations for genome-dependent SAPs, transcription-dependent ASs, and posttranslational-dependent PTMs is quite remarkable. While PTM determination depends upon the sensitivity of protein analytics, SAP and AS detection have virtually no limitations in sensitivity and are actively accumulated via large-scale projects [13]. Despite such differences, all of the technologies have synchronously acquired saturation levels, indicating balance between data derived from using standard protein-chemistry techniques (accumulated over the last 50 years) and data derived from high-throughput next-generation sequencing (NGS).

For estimating the potential number of proteins, three different cases of combination of PTM, SAP, and AS events were considered (see (1)–(3)). Combinatorial variations were ignored, since there are no systematic experimental data describing the cooccurrence of various modification types in the protein species. This is just one of the possible ways for solving the problem of how to estimate a potential number of proteins based on the data of protein variance that has already been accumulated on the postgenomic knowledge bases. Equation (1) assumes that PTMs appear exclusively in the canonical sequences of proteins, but not in splice variants. Equation (2) assumes that PTMs and SAPs can occur both in proteins encoded by canonical sequences and in splice variants. Equation (3) assumes that all modification types (AS, SAP, and PTM) occur independently. Hence,

(1) Nps=N∗ASav+SAPav+PTMav,

(2) Nps=N+AS∗SAPav+PTMav,

(3) Nps=N∗ASav∗SAPav∗PTMav,

where Nps represents the number of protein species, *N* represents the total number of protein encoding genes, AS is the number of species produced by alternative splicing, ASav is the average number of splice variants per one protein encoding genes, SAPav is the average number of nsSNPs, and PTMav is the average number of PTM events per one protein encoding gene.

Generally, SAPs are predetermined at the DNA level, and AS arises from modifications at the mRNA level, while PTMs occur at the protein level. These three processes cannot be viewed as independent events, given that there is an intrinsic relationship between the processes of gene expression, transcription, and translation, aimed at regulating and preserving a cell. Furthermore, enriching MS/MS searches through a database containing all possible combinations of protein variations would lead to combinatorial collapse, despite the type of approach used [14].

The neXtProt (ver. 2015_06) search for protein AS modifications revealed 21,921 AS variants in 10,519 protein-coding genes (2.1 ± 0.1 variants/gene, including one canonical sequence). The greatest number of modified forms (434,398, without cancer-related items derived from the COSMIC cancer mutation database [15]) was due to the emergence of SAPs resulting from nsSNPs in 18,986 protein-coding genes (22.1 ± 3.9 variants/gene). PTMs added 6.6 ± 0.8 modified proteins/gene (94,036 PTMs in 14,006 protein-coding genes). Applying these numbers to the equations (*N* = 20,043), we estimate that in humans there exist 0.62 or 0.88 or 6.13 million protein species.

The above results were matched to the data on AS- and SAP-derived variances obtained from our NGS results of liver tissue transcriptome profiling [16–18]. According to NGS results, the average number of detected splice variants was 1.3 per protein-coding gene (or 2.3 per gene including canonical variant), which is comparable to neXtProt data. The average number of SAP-containing proteoforms was ~1.4 per one gene, so much lower than that calculated from neXtProt data. These differences relate to the fact that neXtProt provides information from many different experiments (“aggregate human population”), while specific NGS data indicates SAP events for an individual sample or tissue (individual variances).

As proteomic knowledge bases consolidate information regarding protein variability in the human population, several million different proteins will ultimately populate the “aggregated” human proteome. To decipher variability inherent in predicting proteome space for an individual, more precise estimation of the numbers of AS- and SAP-contained proteins can be achieved using results of transcriptome profiling of specific tissue samples.

## 3. How Many Protein Species Are Detectable Today?

According to the Plasma Proteome Database (ver. 06_2015) [19], 10.5 thousand blood-plasma proteins have been detected and less than 10% (1278 of 20,043 human proteins) have been measured in a quantitative manner. The primary issue concerning experimental validation of existing sets of theoretically predicted proteins is the limit of analytical sensitivity of proteomic technology. Analytical sensitivity is determined by instrument-dependent detection limit and biomaterial-dependent dynamic protein concentration ranges. Blood plasma is a complex mixture with a dynamic range of protein concentrations varying by >10 orders of magnitude [20], while the protein concentration range of tissue or cell lines is within seven orders of magnitude [21]. The challenge is in detecting low- and ultralow-abundance species with concentrations <10^−12^ M in the presence of high-copied protein molecules at concentrations >10^−6^ M [22].

Assuming the ultrasensitive capacity of oligonucleotide analytics, it is instructive to consider that transcriptome research results are often determined based on copies of RNA molecules rather than concentrations [23]. Operating at low- (<10^−12^ M) and ultralow (<10^−15^ M) concentrations of proteins implies that quantifying protein in copy numbers rather than in concentration units enables comparison of transcriptomic and proteomic results [24].

Proteins are commonly quantified in the proteomics field [25] by the concentration in the biological sample, *C*, reported as mol/L (molarity, M). The corresponding number of protein copies, *N*, in 1 L can be calculated out of concentration units as follows:

(4) N=C∗VRA,C=mMw∗V,

where *R*_*A*_ represents the reverse Avogadro's number, 10^−24^ M [26], *V* represents the sample volume, *m* represents the protein content, and *M*_*w*_ represents molecular weight of the protein.

Formulas (4) address the major challenge of proteomics: shift from concept of the concentration units to counting single biomacromolecules in a sample (tissue) [27].

The triple-quadrupole mass spectrometer makes it possible to achieve 10^−14^ M [28, 29] sensitivity for targeted proteins [30]. The sensitivity of SRM protein detection can be further increased up to 10^−16^ M by irreversible chemical binding proteins from large volumes of biological samples [31] (it is not intended to state that all proteins measured were determined with such sensitivity; results of measurements can vary by several orders of magnitude due to different physicochemical properties of proteotypic peptides).

In the context of proteome width, the targeted approach is limited by the need to measure only proteoforms exhibiting* a priori* assumption of proteotypic peptides, which correctly resemble PTM, SAP, or AS events. In contrast to shotgun MS, SRM cannot discover new, unexpected protein species [32]. Possibilities of top-down and bottom-up MS approaches to address the microheterogeneity of the human proteome were described earlier [33]. Targeted SRM is readily available for detecting SAPs in association with disease, including obesity/diabetes [34] and cancer [35]. For example, SRM/MRM method was applied to measure the quantities of splice forms: three isoforms for transforming growth factor were measured by SRM at concentration level of 10^−11^ M in mouse plasma and human saliva [36]. Another example, osteopontin isoforms, was measured using the SRM assay and revealed that level of isoform was significantly higher for non-small cell lung carcinoma compared with the control group (7*∗*10^−10^ versus 30*∗*10^−10^ M) [37]. The application of targeted MS for the detection of PTMs was illustrated for protein glycosylation: N-glycosides were detected in human plasma at a sensitivity level of 10^−11^ M [38] and ubiquitination [39]. From these pilot studies, it follows that the vast majority of predictable proteoforms seem to be present in the concentrations below limit of detection. Further increase in sensitivity of analytical methods is important to uncover diagnostically relevant proteoforms in human biosamples.

Since it was shown that the set of proteins encoded by any human chromosome constitutes a representative portion for the whole human proteome [40], high-, medium-, and low-copied protein species can be evaluated by sampling master proteins encoded by a single chromosome. As an example of a chromosome-centric proteomic map, we uploaded data from PASSEL [41] (PASSEL IDs: PASS00278, PASS00276, PASS00092, and PASS00742) obtained for master proteins encoded by chromosome 18 [16, 17]. These proteins were measured in three types of biomaterial, including human plasma, liver samples, and HepG2 cells. The measurements were conducted according to Tier 3 (exploratory studies) guidelines [42] using the double targeted strategy, which combines chromosome-centric approach with bottom-up SRM mass spectrometry [43].

**Figure 1:**
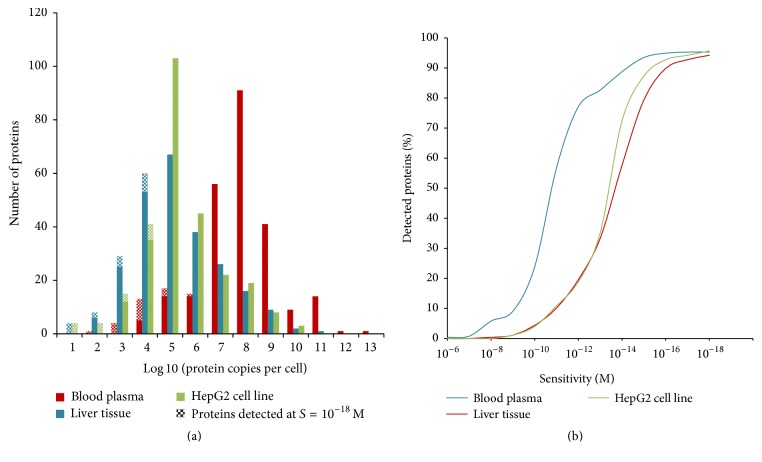
(a) Distribution of the copy numbers of master proteins of chromosome 18 normalized per single HepG2/liver cell or 1 *µ*L of plasma. (b) Share as a function of the detected proteins (in % to the total number of chromosome 18-coded proteins) and the analytical sensitivity.

A bell-shaped distribution histogram for master proteins encoded by chromosome 18 was observed (Figure 1(a)) revealing median of 10^8^ copies per 1 *µ*L of blood plasma and 10^5^ copies per liver/HepG2 cell. The ascending portion of the curve reflects high- and medium-copied proteins, whereas the descending portion may be explained by either diminished proteome diversity in a biological sample or more probably the notion that the proteins cannot be detected due to low sensitivity of the analytical methods [44]. Interestingly, after increasing the sensitivity of the analytical method from 10^−14^ M to 10^−18^ M by irreversible binding of analytes [30], 14 additional low-copied protein species (<10^5^ copies per cell or per 1 *µ*L of blood plasma) were gained and quantitatively measured, with at least two proteotypic peptides in each type of biomaterial (see shaded areas in Figure 1(a)). According to the results, there are much more high-abundant protein species in the plasma as compared with the liver or HepG2 cells. It is, therefore, likely that the difficulty in identifying ultralow-copied proteins in plasma is related to the high dynamic concentration range of plasma proteins [22].

To demonstrate the proteome depth, the number of copies of a master protein in a biosample was plotted depending on the sensitivity of proteomic technology (Figure 1(b)). The proteome coverage was expressed as percent share of detected proteins to the total number of chromosome 18 genes, which was 276 according to neXtProt data. As shown in Figure 1(b), the distribution curve for the plasma proteins shifts left relative to the curves for the cells. The total number of detected protein species in liver and HepG2 cells increased relative to human blood plasma.

Future successes in human proteome exploration depend upon the ability to use bioinformatics methods to elucidate existing protein species and targeted MS analysis, high-throughput measurement, and high-performance algorithms for* de novo* assembly of protein sequences based on MS results. Furthermore, increasing the sensitivity of analytical technology will enable greater access to ultralow-copied proteins and expand opportunities for detection and analysis. In this context, theoretical prediction of the number of proteoforms (estimation of proteome width) and their distribution across the dynamic range (i.e., proteome depth) is ultimately required for planning the workload for the chromosome-centric Human Proteome Project.

## Abbreviations

AS: Alternative splicing
NGS: Next-generation sequencing
nsSNPs: Nonsynonymous single-nucleotide polymorphisms
SAP: Single amino acid polymorphism.
